# Auditory-induced response in the primary sensory cortex of rodents

**DOI:** 10.1371/journal.pone.0209266

**Published:** 2018-12-20

**Authors:** Atsuko T. Maruyama, Shoji Komai

**Affiliations:** Department of Science and Technology, Nara Institute of Science Technology, Takayama, Japan; Ecole Polytechnique Federale de Lausanne, SWITZERLAND

## Abstract

The details of auditory response at the subthreshold level in the rodent primary somatosensory cortex, the barrel cortex, have not been studied extensively, although several phenomenological reports have been published. Multisensory features may act as neuronal representations of links between inputs from one sensory modality to other sensory modalities. Here, we examined the basic multisensory postsynaptic responses in the rodent barrel cortex using *in vivo* whole-cell recordings of neurons. We observed robust responses to acoustic stimuli in most barrel cortex neurons. Acoustically evoked responses were mediated by hearing and reached approximately 60% of the postsynaptic response amplitude elicited by strong somatosensory stimuli. Compared to tactile stimuli, auditory stimuli evoked postsynaptic potentials with a longer latency and longer duration. Specifically, auditory stimuli in barrel cortex neurons appeared to trigger “up states”, episodes associated with membrane depolarization and increased synaptic activity. Taken together, our data suggest that barrel cortex neurons have multisensory properties, with distinct synaptic mechanisms underlying tactile and non-tactile responses.

## Introduction

The mammalian cerebral cortex is a high-level processing structure that is divided into functionally distinct areas [1–3]. The primary sensory areas can be distinguished from other neocortical areas because they 1) receive numerous thalamic afferents that relay inputs from a particular sensory organ and 2) have an exquisite topographic representation of the respective sensory organ. Physiological investigations of primary sensory areas have therefore focused on representations of sensory information, particularly unimodal stimuli, from specific sensory organs.

The representation and integration of multisensory information have been investigated in higher order “associational” cortical areas and midbrain neurons of the superior colliculus [4–10], which receive multisensory inputs. However, quantitative anatomical studies suggest that primary sensory cortical areas are not strictly unimodal, and direct anatomical connections between primary sensory cortices were reported in addition to the secondary connections between primary areas via subcortical structures [11–14].

In recent studies, multisensory responses have been observed in primary sensory cortical areas, mainly involving audio-visual interactions in the primary auditory or visual cortices [15–17]. Indeed, previous studies have demonstrated that the primary somatosensory area responds to auditory cues during a task involving a combination of sound and tactile signals [18,19]. Even in conditions that do not involve such tasks, the rat barrel cortex shows responses to, or is modulated by, visual stimuli [20,21]. Extracellular recordings in these studies revealed that stimulation from another sensory modality (e.g., auditory or visual) did not evoke significant firing in the primary somatosensory cortex. Extracellular and juxtacellular recordings demonstrated that the responses of neurons receiving sensory information were affected by neural adaptation, integration, and oscillations [22]. Cross-modal responses have also been observed in each cortical area, including the somatosensory, auditory, and visual cortices [23,24]. Furthermore, the sensitivity of multisensory processing was reported to be affected by arousal level [25]. However, it is unclear how inputs of multimodal information to the same neuron are processed in the primary sensory cortices. It is possible that subthreshold responses were too small to be detected extracellularly in prior studies [26–29].

The consideration of such connections between primary sensory areas and their multimodal activity profiles led us to investigate the multisensory properties of barrel cortex neurons. In the barrel cortex, the source of auditory responses could originate from the whisker pad, which may vibrate based on sound-induced air movements. In this study, we examined the basic properties of cross-modal responses in the rodent barrel cortex through occlusion studies, including infra-orbital nerve severance and tympanic membrane destruction. Specifically, our experiment aimed to address three questions: First, what are the sources of cross-modal sensory responses in barrel cortex neurons? Second, what are the properties of responses to stimuli from other modalities and the synaptic mechanisms underlying such responses? Finally, how do “non-primary” responses interact with somatosensory and ongoing activity *in vivo*? Addressing these questions will provide insight into whether the multisensory responses described in this study also occur in the human somatosensory cortex.

## Materials and methods

### Animals and anesthesia

All experimental procedures were approved by the ERASMUS MC animal care ethics committee, the Institutional Animal Care and Use Committee of Max Planck Institute, and Nara Institute of Science and Technology [Approval No. 4 and No. 1701]. All animals were kept in the conventional animal facility under a 12 h light/dark cycle until they were transferred to the laboratory in the daytime for experimentation.

Recordings were obtained from 38 Wistar rats of both sexes, which were raised in groups with cage mates and chosen randomly before experimentation. In addition, recordings were obtained from 44 SPF C57BL/6 mice of both sexes; these data are not included in the analysis or shown in the figures. The principles of the 3Rs were adhered to while performing all animal experiments, and the minimum number of animals necessary for appropriate statistical analyses were examined.

Our quantitative analysis focused on data from rats. All data shown refer to rat experiments unless otherwise specified. Animals were between 21 and 29 days old (average: 23.5 days).

Anesthesia and cell recordings were performed as described previously [30]. In brief, rats were anesthetized with an intraperitoneal injection of 20% urethane (5–20 mL/kg). Body temperature was maintained at 36°C using a heating blanket (Watlow). Several animals died at 37°C, which is the temperature at which animals were typically housed. From our experience, the slightly lower temperature setting was safe and effective. We conjectured that the heating pad used for this experiment yielded a body temperature higher than the set temperature. Due to blood circulation that facilitates homeostasis, the temperature of the brain is relatively resistant to changes in body temperature. Indeed, it was possible to record normal sensory responses, and the animals awakened after a couple of tens min of recording. The amplitude of the post-synaptic potential (PSP) was 10.3 ± 0.7 mV on average, and the resting potential was 72.8 ± 1.8 mV. This indicated that the PSP did not reach the threshold to fire even after a single sensory stimulus, whereas it could have occasionally reached threshold due to fluctuations. In addition, most *in vivo* patch clamp recordings in the barrel cortex revealed low firing rate of neurons in line with the literature. Brecht, Roth, and Sakmann reported that the average spontaneous action potential (AP) activity was low (0.068 ± 0.22 APs s^-1^) [30]. They observed low firing rate of L2/3 neurons of the barrel cortex even after principal whisker (PW) deflection was induced. In agreement with these findings, Lee, Manns, Sakmann, and Brecht reported relatively low firing rate (0.36 Hz) in whole cell *in vivo* recordings [31]. A 1-mm diameter hole was drilled into the skull, 5.5 mm lateral to, and 2.5 mm posterior to bregma. The dura mater was removed with a 30-gauge injection needle tip. The exposed cortex was covered with HEPES-buffered artificial cerebrospinal fluid (ACSF) solution. The depth of anesthesia was monitored by pinch withdrawal, eyelid reflex, corneal reflex, respiration rate, and vibrissae movements. Under the recording conditions employed, pinch withdrawal and vibrissae movements were usually absent, but weak eyelid and corneal reflexes were observed. The depth of anesthesia during recordings was generally lower than during the initial surgery. When both vibrissae movements and withdrawal reflexes started to appear during the experiment, an additional dose of urethane (20% of the initial dose) was injected.

### Stimuli and whole-cell recordings

Basic recording procedures were performed as described previously [22]. Recordings were made with long taper (up to 2 mm) patch pipettes (Harvard Apparatus, MA, USA: GC150-7.5) with resistances of 4–8 MΩ pulled from borosilicate glass tubing on a Sutter puller (model P-97: Science Products, Hofheim, Germany) in a four-stage pull. Pipettes were filled with [mM]: potassium gluconate 130, sodium gluconate 10, HEPES 10, phosphocreatine 10, MgATP 4, Na_2_ATP 2, GTP 0.3, and NaCl 4, with 0.4% biocytin at pH 7.2. To prevent tip occlusion, light pressure (20–30 kPa) was applied to the pipette interior during insertion into the brain. Conventional voltage clamp techniques were used to locate cells, and data were recorded in the bridge-balance mode [32,33]. All data were recorded from layer 2/3 (estimated depth: 58–494 μm; average: 230 μm), and 35 of the 49 cells were located in the barrel cortex. The presumed location, firing pattern (including basic properties such as resting membrane potential and input resistance), and whisker responses were assessed for all recorded cells. Series resistances were between 20 and 85 MΩ. All data were corrected for a +7 mV junction potential. Rather than using a stereotactic apparatus, a head-plate or small bolt was used to fix the head in order to ensure auditory stimuli reached the ear.

All tactile stimulation procedures were performed as described previously [30]. Whisker stimuli were delivered with a piezoelectric stimulator (Physik Instrumente GmbH&CO. KG, Germany). A glass capillary was attached to a piezoelectric bimorph wafer equipped for quantitative single-whisker stimulation [34]. Electrical steps from the piezoelectric device were given as a 10–90% rise time of 1 ms to elicit activity. The deflection point of the whisker was set between 5–10 mm from the base of the vibrissa, and the deflection to vibrissa was then given backwards by 1 mm (approximately 6° deflection angle) for 200 ms at a frequency of 1 Hz. All cells were stimulated with piezoelectric stimulators (mainly on the whiskers in C-E row). All displayed data refer to this type of stimulation. For tactile and auditory response PSPs, we classified the largest membrane depolarization at the initial 200 ms and 500 ms time points after stimulus onset (identified as the first timing of the rising point after sensory stimulation) in averaged traces, respectively. For PSP latency measurements, we determined the time point, after whisker deflection onset, at which the PSP reached 5% of its peak amplitude. For auditory stimulation, we played 30 ms of rectangular white noise from a customized earphone system or a 5-cm diameter speaker (50 Ω, 1 W) placed 5–10 cm away from the ear of the animal.

**Fig 1:**
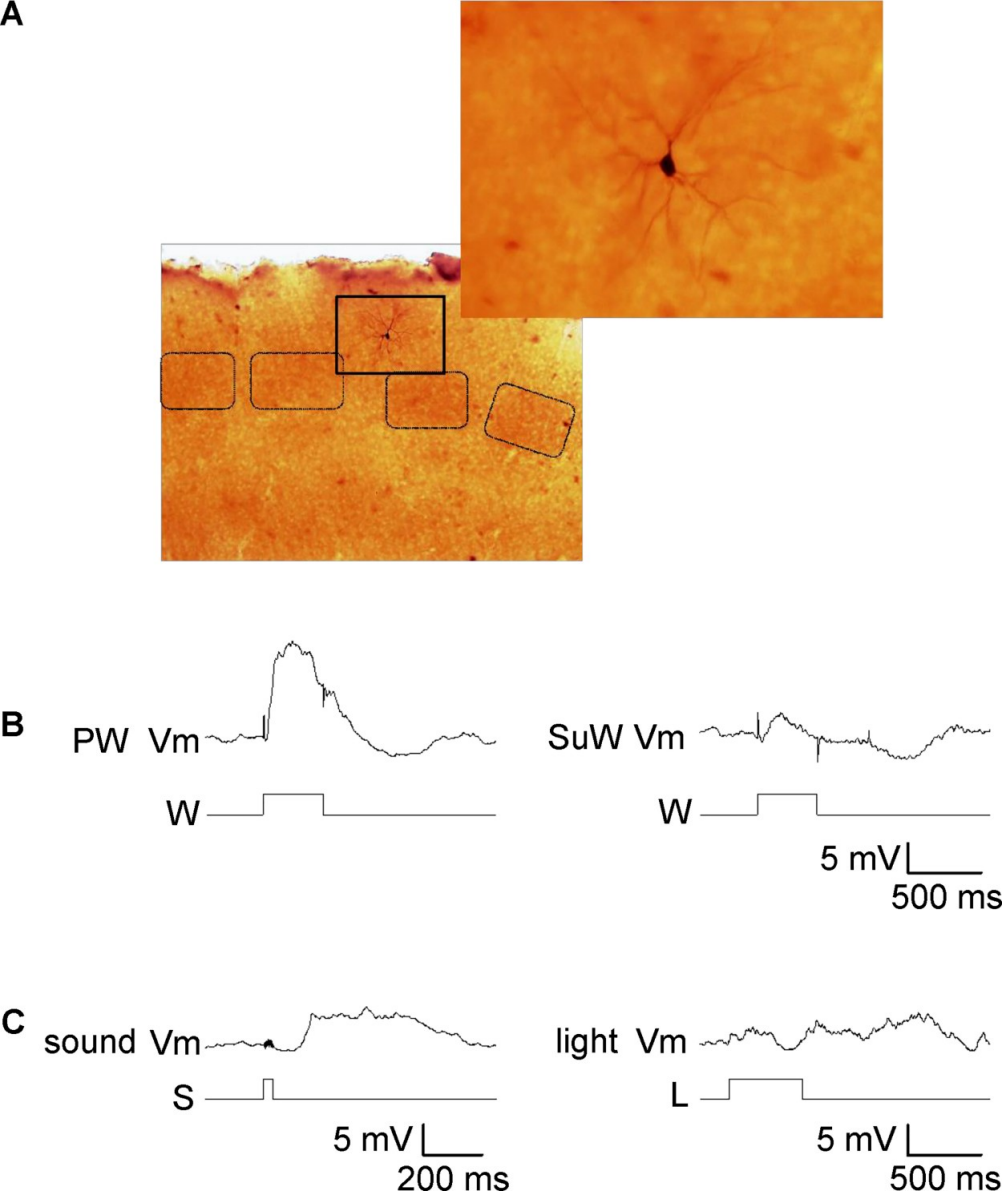
Typical tactile and non-tactile responses of a neuron in layer 2/3 of the barrel cortex. (A) An example of a recorded pyramidal neuron stained with cytochrome C and biocytin after *in vivo* recording that was located above a barrel. A lower-magnification image of the cortical field is shown on the top right. (B) Tactile responses to principle (PW) and surround whisker (SuW) deflection recorded from the neuron shown in (A). Stimulation timing with a piezo apparatus for these recordings is indicated below the traces. (C) Typical auditory and visual responses in the barrel cortex recorded from the same cell as in (B). The timing of auditory stimulation (white noise) and visual stimulation (LED red flash) is indicated below the traces. Auditory, tactile, and visual stimulations are indicated by S, W, and L respectively.

Controlled auditory stimulation was applied in a soundproof booth. We applied sound stimuli via a custom-made silicon glue earphone and a calibrated TDT audio synthesizer system (Tucker-Davis Technologies, Alachua, USA). Noise bursts were applied at 35, 50, 65, and 80 dB ranging from 8 to 12 kHz with a 5 ms rise time. Pure tones of 80 dB with a 100-ms duration were applied at 1 to 30 kHz. Auditory stimulation was applied with 10 trials that were repeated at an interval of 5 s for each frequency. Stimulation amplitude was ramped from 0.1–10 V (80 dB at 10 V, Fig 1) with 10 or 20 kHz pure tones. Sound pressure level was calibrated with detectable sounds recorded with a digital sound level meter (Benetech) and given voltage to the audio synthesizer system.

The infraorbital nerve (ION) cut was performed. Cells in the recorded area were then checked as indicated by the whisker responses. Facial hair was shaved off to strip the skin behind the whisker pad. A small portion of skin in that location was then cut to reveal the ION so it could be severed. Once the nerve was ready to be cut, the cell in S1 was patched, and the whisker and sound responses from one cell were recorded before and after the nerve was cut in the same area.

For the tympanic membrane rupture, the cell in S1 was continuously patched to examine whisker and sound responses before and after the tympanic membrane was cut with a 30G needle. The success of membrane rupture was confirmed by hearing and feeling it break. The sound responses from the same cell were continuously recorded after breaking the tympanic membrane.

An LED flash was applied to the animal as a visual stimulus directly in front of the eye ball. The strength of the LED was determined by eye to provide the animals sufficient brightness by which its flashing could be easily detected even with eyes closed.

After whole-cell recordings, animals were kept deeply anesthetized with an additional dose of urethane. The animals were then perfused transcardially with 0.1 M phosphate buffered saline (PBS) followed by 4% paraformaldehyde. Coronal sections from the obtained brain tissue were cut and then stained with cytochrome C oxidase [35] to visualize the cortical layers and barrel structure. The slices were processed using the avidin-biotin-peroxidase method [36] and mounted on slides with Mowiol mounting medium (Clariant, Sulzbach, Germany) to confirm that the recorded cells were in the barrel cortex.

### Outcomes

The primary outcome of this study was the subthreshold effect of auditory stimulation on the activity of barrel cortex neurons. The secondary outcome was the origin of multisensory responses in the rodent somatosensory cortex.

### Statistical analysis

Statistical evaluation of sensory responses was performed with Student’s t-test using R software for most of the recorded data. An ANOVA was used to analyze data for sensory responses to sounds of various frequencies and amplitudes. Post-hoc analysis was conducted using Tukey’s comparison to identify the difference between the conditions. Comparisons were made between the bottom-to-peak amplitude within 200 ms just before a sensory stimulation and at 200 ms after sensory stimulation. Statistical significance is indicated herein as * and **, which correspond to P < .05 and P < .01, respectively.

## Results

All animals met the health care criteria (including lie of hair, daily behavior, etc.) before and during our experiments. The depth of anesthesia was monitored based on pinch withdrawal, eyelid reflex, corneal reflex, respiration rate, and vibrissae movements. In rats, respiration rates were usually between 70 and 100 breaths/min, indicating that the depth of anesthesia varied in the animals around anesthetic state III-3 [37]. To examine multisensory representations and cross-modal integration in the barrel cortex, *in vivo* whole-cell recordings of barrel cortex neurons were performed and multisensory responses were examined. All recorded sensory responses were analyzed. The location of the recorded cells was subsequently analyzed using cytochrome C and biocytin staining [35,36].

### Tactile and non-tactile responses in the barrel cortex

Fig 1 shows representative responses of a barrel cortex neuron (Fig 1A) to tactile, auditory, and visual stimulation. Stimulation of the PW resulted in a PSP with an amplitude of 10.3 ± 0.7 mV (n = 17; Fig 1B), whereas stimulation of a surrounding (neighboring) whisker evoked a smaller PSP (6.3 ± 0.8 mV, n = 15; Fig 1B). This neuron also responded to auditory stimulation, with PSPs of up to 5 mV (Fig 1C left), while no obvious response to visual stimulation was detected (Fig 1C, right).

**Fig 2:**
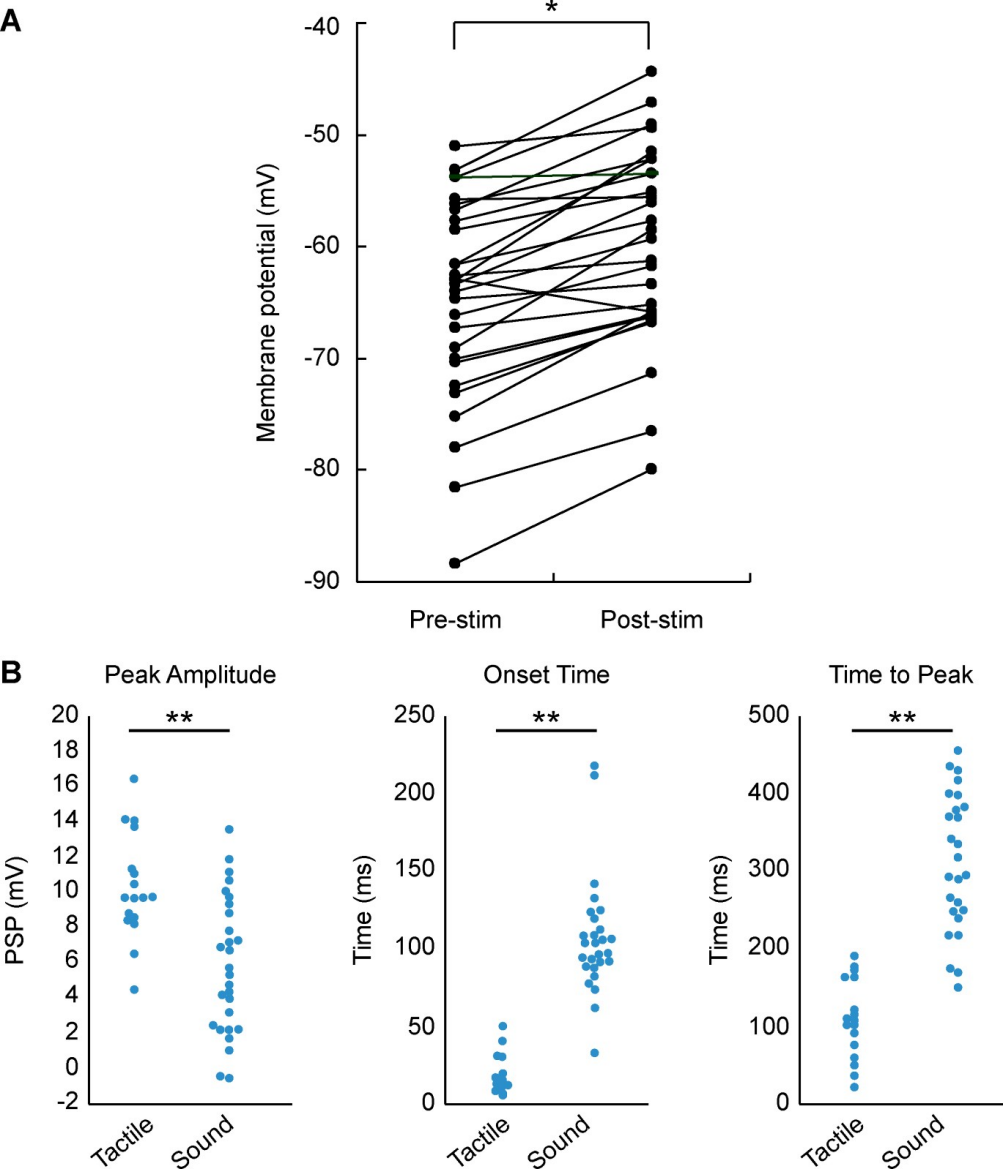
Properties of auditory and tactile responses. (A) Comparison of average membrane potential between pre- and post-stimulation for sound in each cell. (B) Peak amplitude, onset latency, and peak latency of auditory and tactile responses (n = 27 and 17 for sound and tactile responses, respectively). Filled and open circles indicate the values of auditory and tactile responses, respectively. Asterisks indicate significant differences between groups (*P < 0.05, **P < 0.01).

Auditory and tactile responses were examined in 68 neurons in total (38 in rat barrel cortex and 30 in mouse barrel cortex), as determined by the recording depth and location. A subset of cells was located in the supragranular layer of the barrel cortex. Barrel cortex cells responded to a wide variety of auditory stimuli such as handclaps, loud voices, clicks, and pure tone stimuli. Our quantitative analysis focused on the effects of 30 ms broad-band noise bursts. As expected, most barrel cortex cells (65 of 68 cells; 96%) showed statistically significant tactile responses upon comparison of amplitudes between 200 ms pre- and post-stimulus bottom-to-peak amplitude (see Materials and Methods). Auditory responses in the barrel cortex were robust (P = 2.8 x 10^−8^ in population data; Fig 2A), and 63% of tactile-responsive cells (41 of 65 cells) showed significant auditory responses. Auditory responses differed from tactile responses, exhibiting a smaller amplitude with longer latency and duration (Fig 2B). Sound-evoked responses reached approximately 60% of the PSP amplitude of whisker-evoked responses (5.9 ± 0.7 mV vs. 10.3 ± 0.7 mV for averaged responses, P = 1.9 x 10^−4^), but showed a significantly longer onset latency (106 ±7 ms vs. 18 ± 3 ms, P = 1.2 x 10^−11^) and a slower time to peak (319 ± 19 ms vs. 110 ± 12 ms, P = 2.5 x 10^−10^). Obvious visual responses evoked by a bright red LED were undetectable due to their small amplitude (< 4 mV) and variable (300 to 1000 ms) peak time. In mice, auditory responses had similar properties to rats but were larger in average amplitude (12.4 ± 1.2 mV).

**Fig 3:**
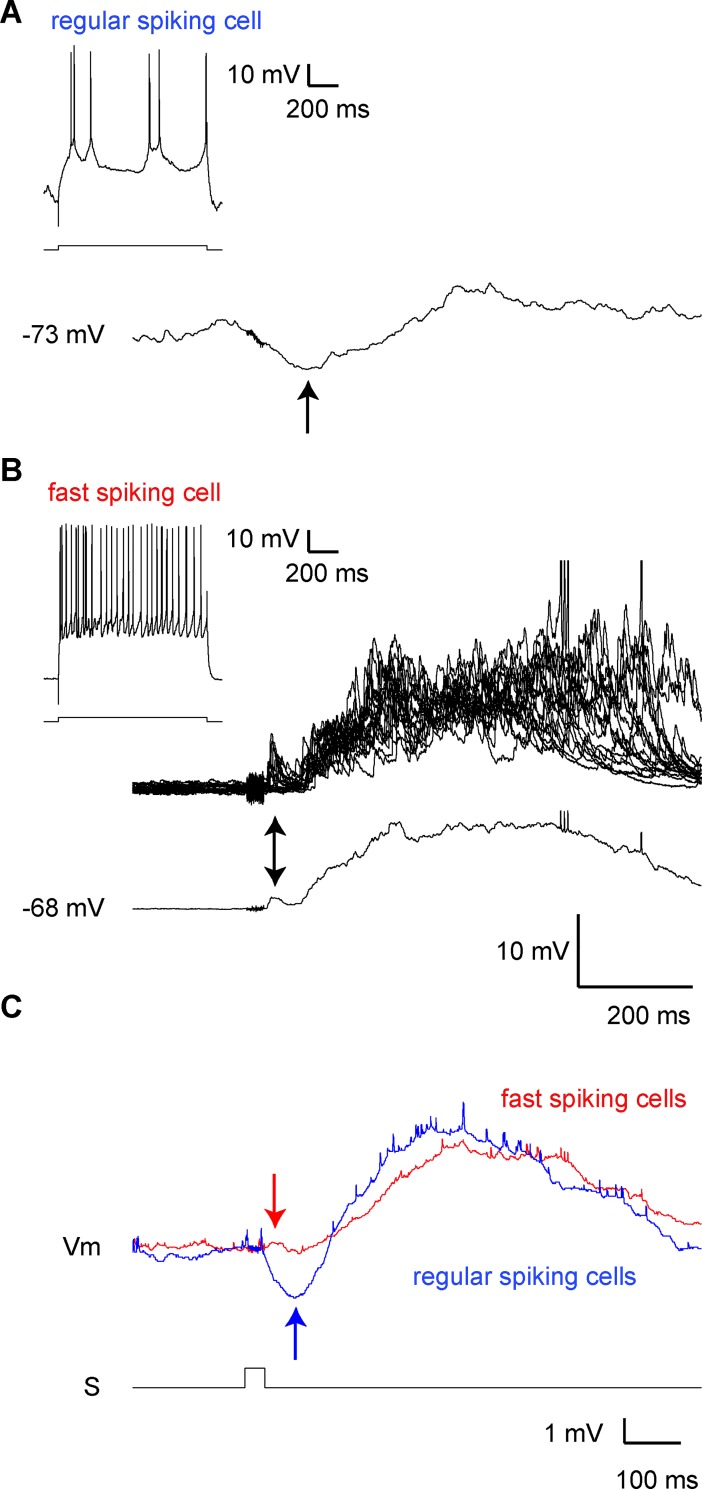
Comparison of auditory responses between regular-spiking (RS) and fast-spiking (FS) cells. (A) An example of the auditory response of an RS cell in layer 2/3 of the barrel cortex. Responses for 20 repeated trials were averaged. Typically, a hyperpolarizing response (arrow) was followed by depolarization. Firing pattern by square-wave current injection is shown in the upper-left. (B) Twenty consecutive responses (upper) and their average (lower) in a representative fast-spiking cell. Two peaks were observed in most FS cells, corresponding to fast (arrow) and slow components. (C) Superimposed average response of RS and FS cells. The fast component in the FS cell (red arrow) showed a fast peak compared to the hyperpolarization profile of the RS cell (blue arrow). S, auditory stimulation.

Auditory responsive neurons in the barrel cortex included both regular-spiking (RS) and fast-spiking (FS) cells. FS cells were identified by their shallower membrane potential (~ -60 mV), lower input resistance, and firing frequency over 100 Hz. The proportions of these cell types were similar (62%, 33 of 53 RS cells vs. 67%, 8 of 12 FS cells). RS cells exhibited hyperpolarization followed by depolarization in response to auditory stimulation (Fig 3A), whereas FS cells showed two depolarization peaks (Fig 3B). The depolarizing phase in RS cells and the slow component of depolarization in FS cells had a similar time course, although the fast component in FS cells reached peaks faster than did the hyperpolarization in RS cells (Fig 3C).

### Sound responses are mediated by the auditory system

**Fig 4:**
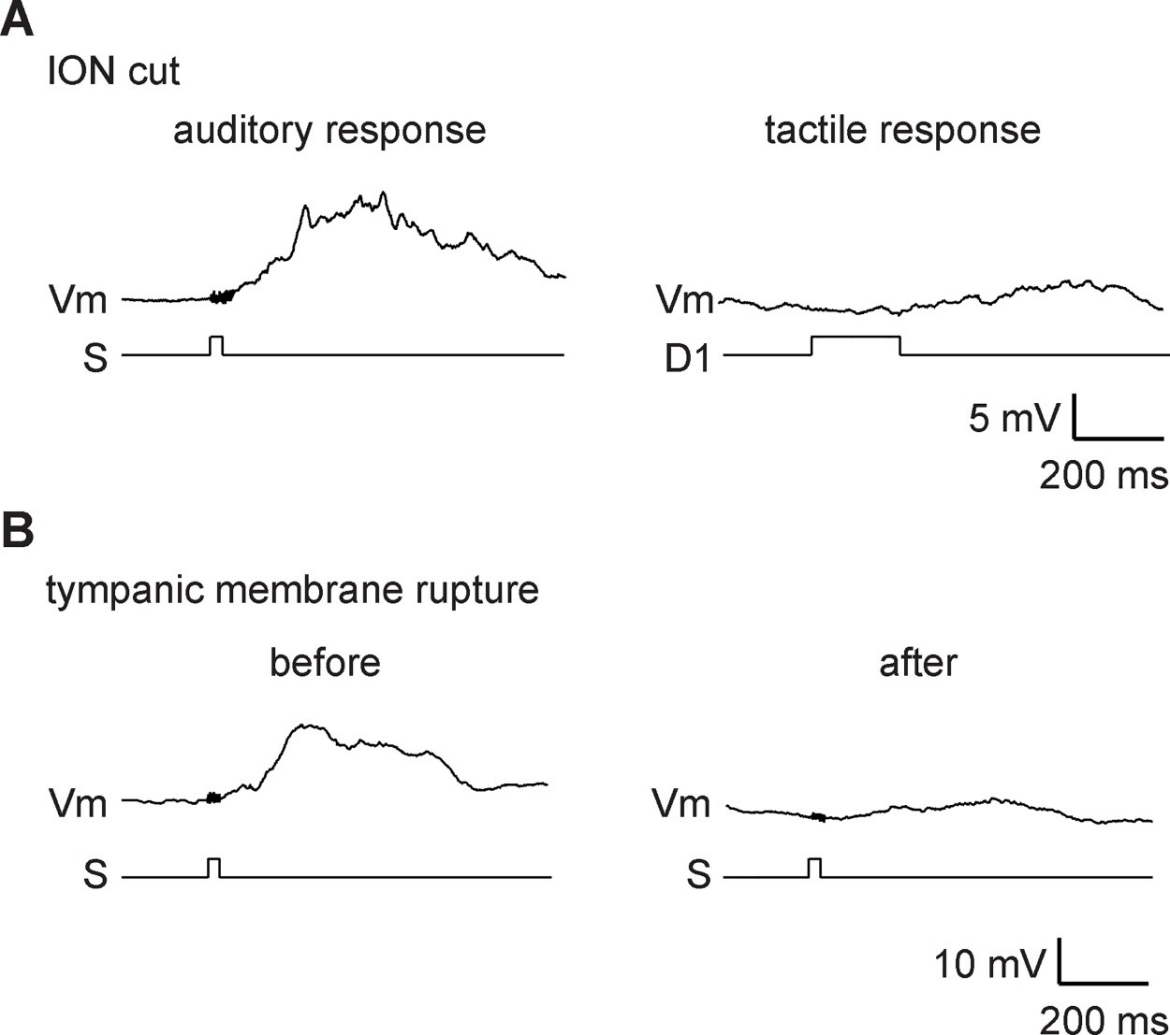
Auditory responses originate from the auditory system, not vibration of facial whiskers. (A) Auditory responses remained intact (5.8 ± 1.0 mV: n = 3) after the infraorbital nerve (ION) had been cut, although tactile responses were abolished. A principal whisker response is shown on the right. (B) Auditory response was eliminated after breaking of the tympanic membrane. Auditory and tactile stimulations are indicated by S and D1, respectively.

**Fig 5:**
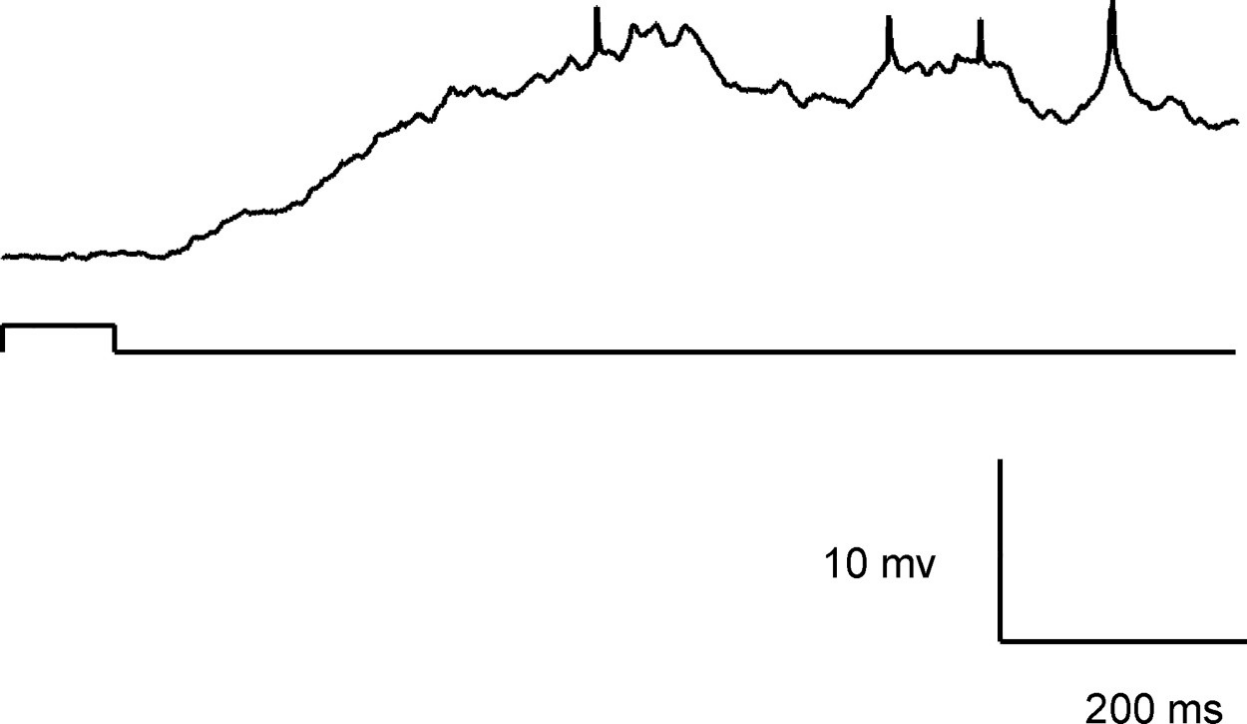
Auditory responses in the left hemisphere barrel cortex elicited by acoustic stimulation of the right ear with a small earphone. Auditory response was elicited even if a short white noise (similar to that in Fig 1) was given specifically at the contralateral side through a small earphone.

Loud sounds evoke twitching of the whiskers; this triggers a subsequent somatosensory response. A series of control experiments was thus performed to identify the pathway responsible for acoustic stimuli-evoked synaptic responses in the barrel cortex. The ION, the branch of the trigeminal nerve that innervates the whisker pad, was cut in four experiments. Subsequently, sound responses could still be evoked, even though whisker responses were completely abolished (Fig 4A). Auditory responses could also be evoked when auditory stimulation was presented via an ear phone (Fig 5). However, auditory responses were markedly diminished (down to 12.7%) following destruction of the tympanic membrane (three experiments, Fig 4B). These findings suggested that auditory responses did not result from airborne whisker vibrations and were indeed mediated by hearing.

### Frequency and amplitude tuning of auditory responses

**Fig 6:**
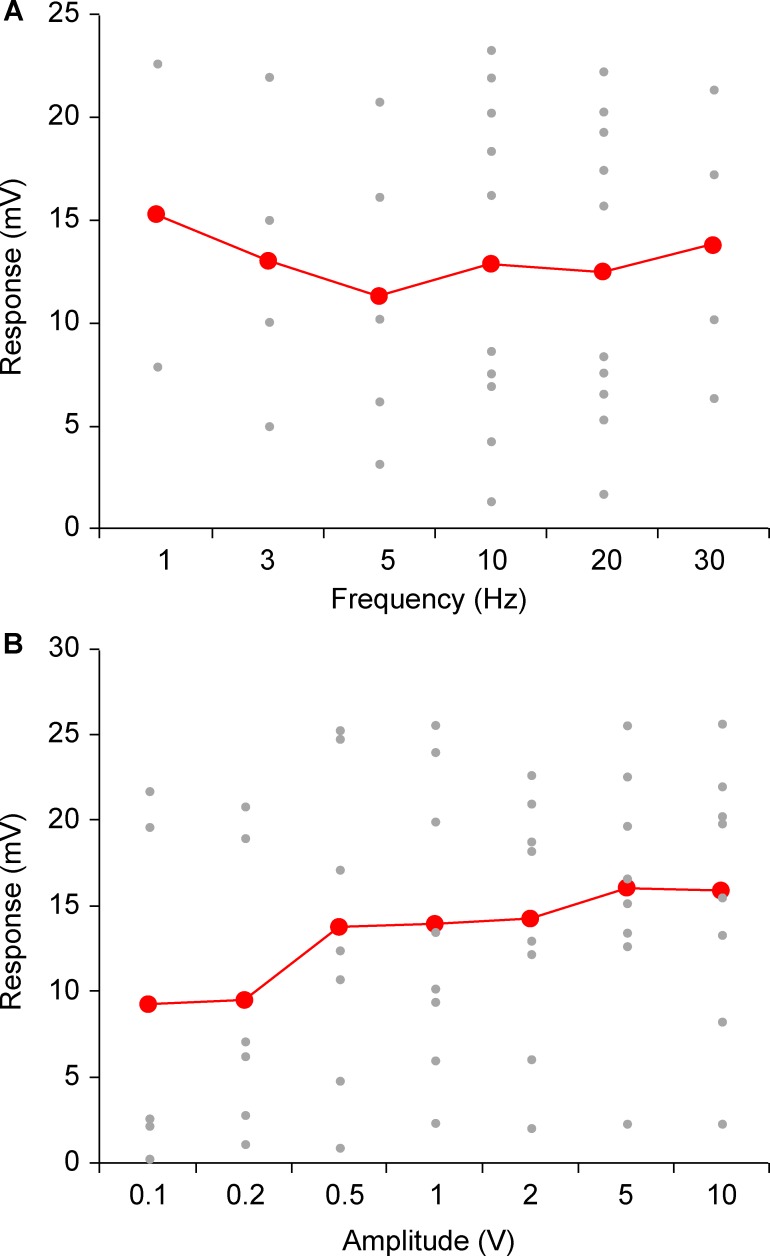
**Responses to sounds of various frequencies (A) and amplitudes (B).** The gray dots show averaged responses of each recorded neuron. Responses of 10 repeated trials were averaged for each neuron. The red line shows averaged responses of all recorded neurons. In (B), sensory evoked excitatory postsynaptic potential amplitudes are shown for the operational signal amplitudes that were input to the earphone. The relationship between signal amplitude (V) and sound pressure level (dB) is shown in S1 Fig; 10 V corresponds to 80 dB sound pressure level.

**Fig 7:**
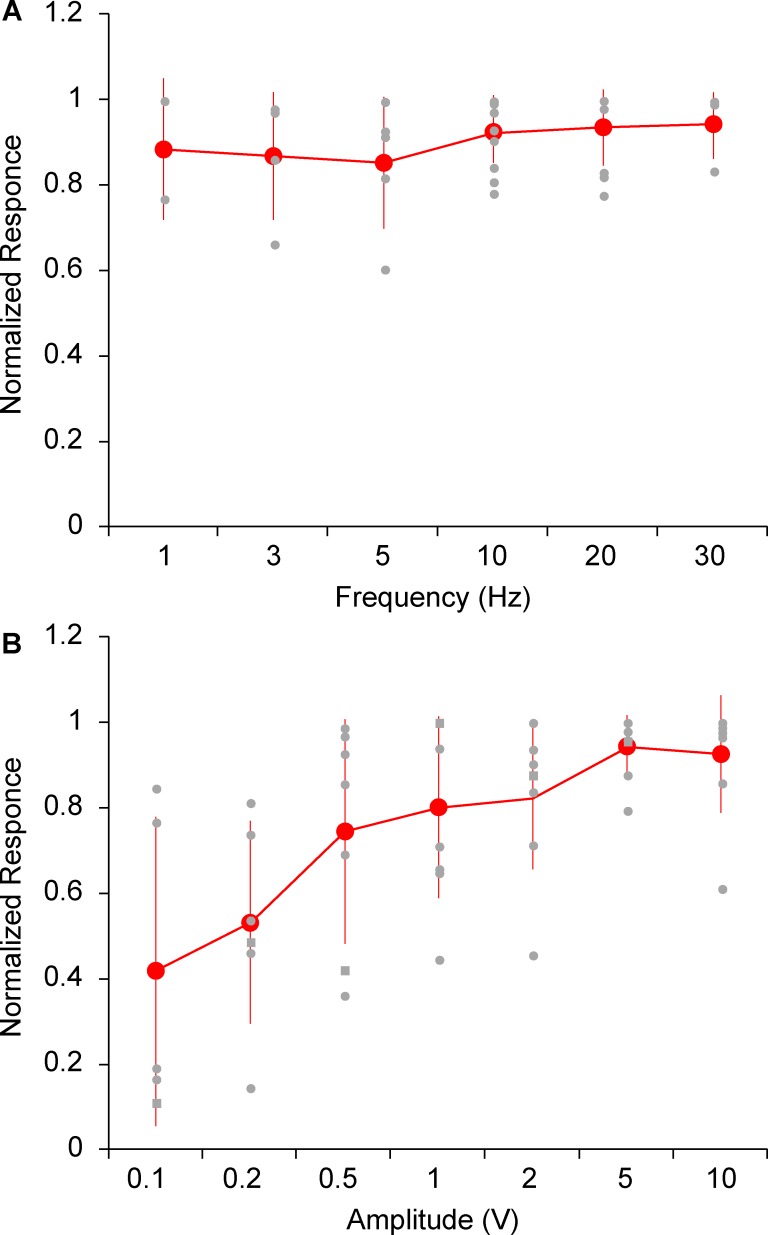
**Normalized responses to sounds of various frequencies (A) and amplitudes (B).** The gray dots show the responses of each recorded neuron. The average response of 10 repeated trials was normalized to the maximum. The red line shows averaged responses of all recorded neurons, and the error bars indicate standard deviation. In (B), amplitudes are shown as the signal amplitudes input to the earphone. The relationship between signal amplitude (V) and sound pressure level (dB) is shown in S1 Fig; 10 V corresponds to 80 dB sound pressure level.

To quantitatively assess the tuning properties of auditory responses, 14 additional neurons in the barrel cortex were recorded. Auditory stimuli of 100 ms were applied in a calibrated set-up at various frequencies and sound pressure levels via an earphone in a soundproof booth. Responses to each stimulus were evaluated as the average of 10 repeated trials, including failures. Barrel cortex neurons were either unresponsive or weakly responsive to sound frequency in the range of 1–30 kHz. The average responses of total cells (n = 10) to each stimulus were similar (11.4–5.3 mV, Fig 6), but the variation between cells was relatively high (SD = 7.2–10.5 mV) as some neurons showed large responses to each stimulus, while others showed small responses with a high failure rate (Fig 6A). Therefore, responses were normalized using the maximum response of each cell. Averages of normalized responses were 0.85–0.94 (SD = 0.08–0.16, Fig 7A). There was no significant difference between frequencies (P = 0.67). In the stimulation amplitude range of 0.1–10 V (80 dB at 10 V, S1 Fig), average responses of total cells (n = 8) of each stimulus were 9.3–16.0 mV (SD = 7.1–0.5 mV, Fig 6B). There was a tendency for large amplitudes to evoke large responses. Averages of the normalized responses were 0.42–0.94 (SD = 0.07–0.36). A significant increase of responses with an increase in stimulus amplitude was observed (P < 0.001, Fig 7B). The normalized responses at 0.1 and 0.2 V were significantly smaller than that at 10 V, the maximum amplitude (P < 0.05).

### Interaction between somatosensory and auditory information

**Fig 8:**
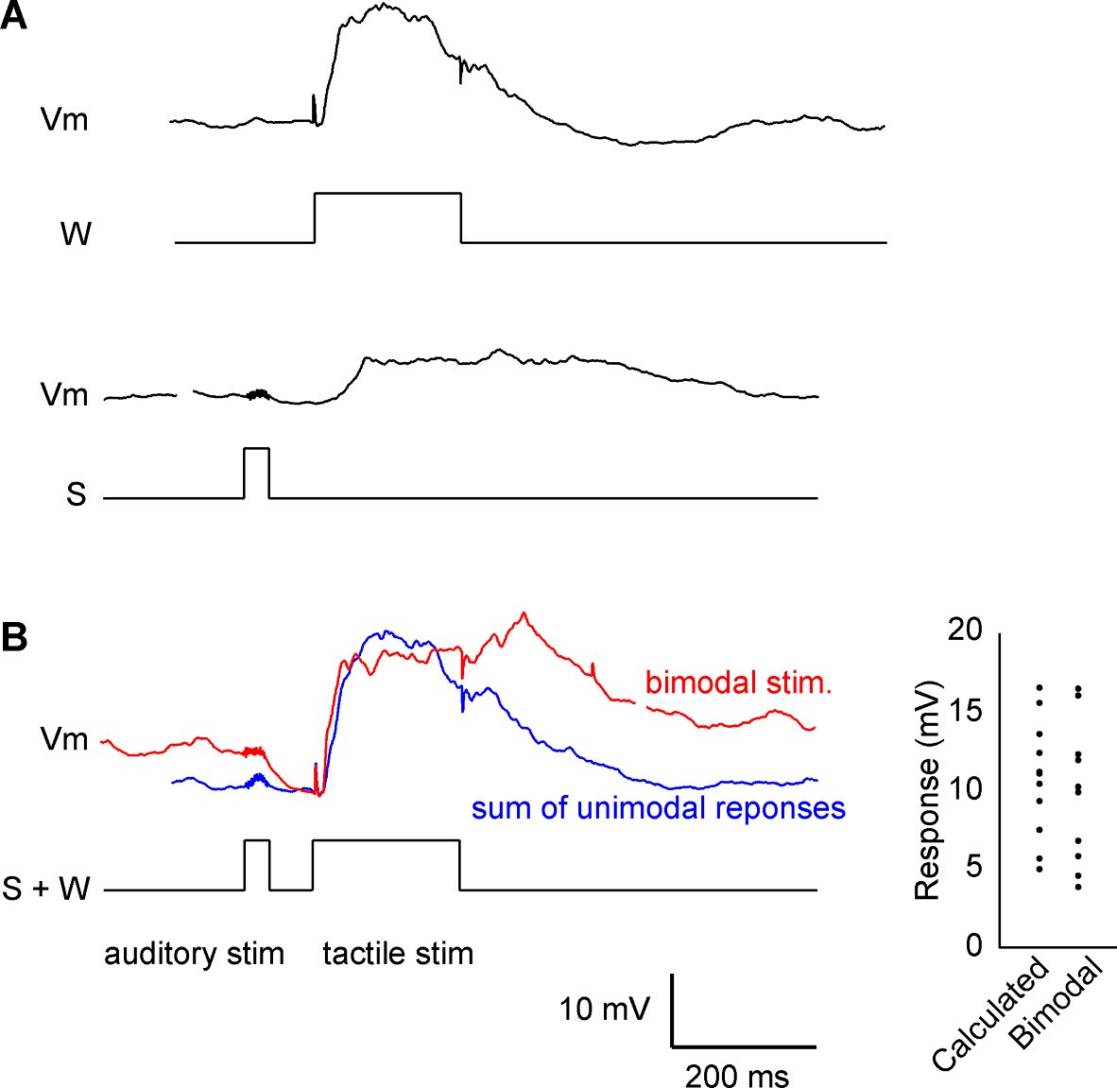
Interaction between auditory and tactile responses. (A) Unimodal responses in the same cell. A regular spiking cell in the barrel cortex responded to auditory stimulation (S). (B) Auditory and tactile (W) stimulation combined and compared with the sum of both individual responses. Population data are shown in the insert. There was no significant difference between the calculated sum of both modalities and combined responses.

To characterize the interaction between auditory and tactile responses in single neurons of the barrel cortex, both unimodal and bimodal stimuli were applied. To compensate for the latency differences between tactile and auditory responses, tactile stimulation was applied 100 ms after the onset of auditory stimulation. The responses evoked by bimodal stimuli were compared, and the linear sum of both individual auditory and tactile responses was calculated. The ratio of the bimodal response to the calculated linear sum of unimodal responses was 95.3 ± 16.1% (n = 6; Fig 8), indicating that majority of the responses added linearly. These data suggested that auditory and tactile information were processed in parallel in the barrel cortex.

### Interaction of sound responses with ongoing cortical activity

**Fig 9:**
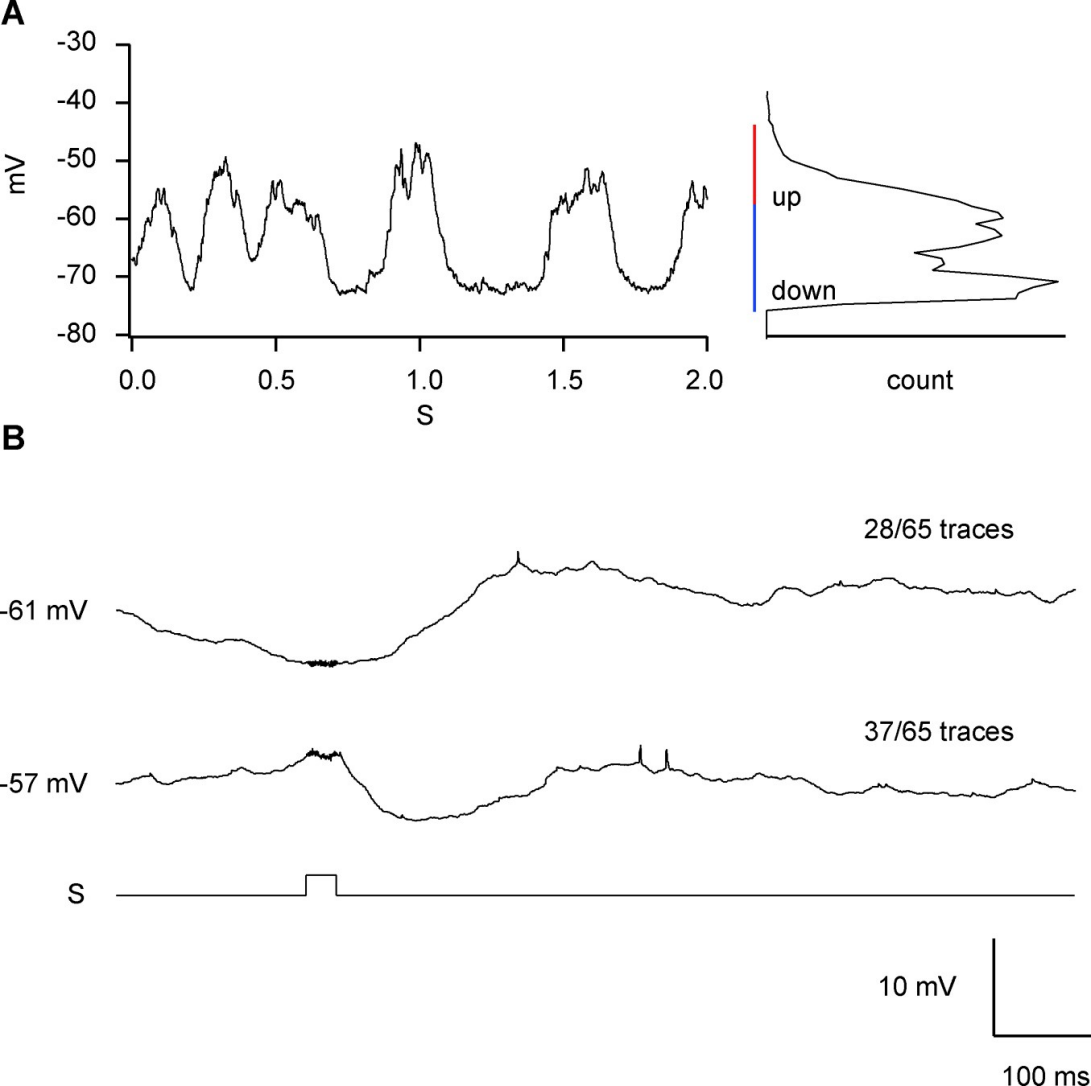
Auditory response depends on up and down states. (A) Intracellular membrane potential clearly shows up and down states. (B) Traces were averaged after classification according to the membrane potential at the time of stimulus presentation. The membrane potential distribution is shown on the right. The red and blue bars indicate the range of distribution of membrane potentials for sorting in (A).

Under urethane anesthesia, the membrane potential of cortical neurons fluctuated by 10–15 mV between depolarized (up states) and hyperpolarized (down states) membrane potentials (Fig 9A). To understand how auditory-evoked responses interacted with ongoing cortical activity, data were sorted into trials based on whether the auditory stimuli were presented in the up or down state (Fig 9B). Auditory-evoked excitatory PSPs were most obvious when the auditory stimulus was presented during a cortical down state (Fig 9B, top). In this regard, auditory responses resembled tactile responses, for which a reduction in response amplitudes during stimulus presentations in the up state has been described previously [38,39]. Concurrently, the initial inhibition of the up-state probability observed in the auditory responses was prominent when auditory stimuli were presented during an up state (Fig 9B, bottom).

## Discussion

In this study, whole-cell recordings were chosen because they enable the recording of subthreshold activity. Robust and wide-spread auditory responses were observed in excitatory and inhibitory neurons of the barrel cortex. Auditory postsynaptic responses reached approximately 60% of the amplitude of tactile responses. Sound responses could be mediated either by the auditory system or be generated by a variety of unconventional pathways and mechanisms. Rats were anesthetized during recording of multisensory responses as sudden sensory stimulation may evoke a startle reflex which has been reported to be sensitive to anesthetics [40,41]. One obvious possibility is that sound can generate somatosensory cortical responses via airborne whisker vibrations [42]. We observed that auditory responses were indeed mediated by the auditory system, and bimodal tactile-auditory responses approximated the sum of unimodal responses.

Auditory barrel cortex responses differed in three ways from somatosensory responses: (1) a longer latency (100 ms vs. 10 ms); (2) a delayed peak response (300 ms vs. 100 ms); and (3) a different sequence of synaptic inputs in a regular spiking cell (inhibition-excitation instead of excitation-inhibition). Cross-modal studies combining tactile and auditory stimulation have reported that barrel neurons evoke more firing in response to a tactile stimulus with sound than to that without sound [28,43,44]. This modulatory effect can be explained by an increased suprathreshold state induced by the summation of tactile and auditory responses, although the auditory response itself is at a subthreshold level that is undetectable with extracellular recordings.

The long latency, delayed response peak, and inhibition-excitation sequence also distinguish barrel cortex auditory responses from the auditory responses observed in the primary auditory cortex [45]. Multimodal responses have been reported in various areas, such as the superior colliculus [46,47] and multimodal cortical areas [6]. Multimodal responses in primary sensory cortical areas in the primary auditory or visual cortex have been observed in recent studies [15–17,48]. However, such studies focusing on the barrel cortex are limited, and only modulatory effects have been reported [20,21,24]. The present study demonstrated that majority of neurons in the barrel cortex had direct subthreshold responses to sound. Anatomically, there are several pathways through which auditory inputs may be sent to the barrel cortex. These include projections from the auditory or multisensory thalamic area [14], corticocortical connections from the primary auditory cortex to the barrel cortex [11,14], and feedback from higher associational cortices to the barrel cortex [49].

Several studies have documented multimodal responses in a fraction of cells from early sensory areas during extensive learning of polysensory stimuli [18,19,50,51]. The results presented here are different as they were obtained from naïve animals that had not undergone such training procedures and demonstrated polysensory responses in majority of cells. The widespread presence of polysensory responses in naïve animals may form a synaptic basis for the more specific forms of polysensory learning observed in earlier studies.

Majority of auditory responses in the barrel cortex were weakly tuned to sound frequency (Fig 7A). There was, however, a significant increase in response amplitudes with increasing sound pressure levels (Fig 7B). The lack of specific tuning and the long latencies of sound responses make it unlikely that auditory barrel cortex responses are involved in the evaluation and interpretation of the auditory properties of sound stimuli. It is more likely that the significance of these responses lies in the altered processing of somatosensory signals for loud stimuli.

Tactile and auditory information is highly relevant for nocturnal animals, including rats [52,53]. Auditory responses may be related to a surprise/alarm value of loud auditory stimuli that can result in startle responses in awake animals. However, it is unclear to what extent this applies to anesthetized animals. Inhibitory effects in the early phase of the auditory response may share a similar mechanism to prepulse inhibition (PPI). PPI is the suppression of the startle response when a weaker stimulus is presented just before the intense startling sound. Both suppression phenomena are elicited by acoustic stimulation and have a peak of approximately 100 ms. PPI is reduced by lesions of the entorhinal cortex, which results in accumulation of dopamine in the nucleus accumbens [54]. It would therefore be worth investigating how nucleus accumbens activity modulates sound responses in the barrel cortex.

Animals may register auditory information as an alarm or remote cue signaling the onset of tactile information that they can subsequently sense in proximity. In a nocturnal environment, animals need to detect the existence of conspecifics and enemies, especially signs of approaching enemies that need to be detected rapidly. Being deprived of vision, sound is likely the first warning the animal can detect, followed by tactile information. The combination of auditory and tactile cues may thus yield effective responses to enemies, both real or imagined, suggesting that such multisensory information processed in the primary cortex may occupy part of an animal’s attention. The combined response of auditory and sensory information may be potentiated after conditioning with an aversive stimulus. However, only additive responses were observed in this study.

Observations related to our findings arise from the analysis of auditory evoked potentials. In many event-related potential (ERP)/electroencephalogram (EEG) studies, a prominent late potential referred to as P300 or P3 has been described. The P300 potential is related to higher order information processing [55,56]. The time sequence of inhibitory and excitatory effects on up-and-down states elicited by auditory stimulation (Fig 9) matches the N1 and P3 potentials recorded by ERP or EEG. Such auditory-evoked potentials have also been described in rats and mice [57,58], and can even be evoked during rapid eye movement (REM) sleep [59]. The mechanisms underlying the P3 potential are being actively investigated [60,61] and may ultimately provide insights into whether the multisensory responses described in this study also occur in the human somatosensory cortex.

## Supporting information

S1 Fig Relationship between operational signal amplitude (V) and sound pressure level (dB). The relationship between amplitude of the operated stimulation and the recorded sound pressure level with the detector is indicated. Abscissa is shown with a logarithmic scale.
(TIF)

S1 File The checklist to report that we follow the appropriate guideline for in vivo experiments by using experimental animals. (PDF)

## References

[1] Olcese U, Iurilli G, Medini P. Cellular and synaptic architecture of multisensory integration in the mouse neocortex. Neuron. 2013;79:579–593. 10.1016/j.neuron.2013.06.010 23850594

[2] Molnár Z, Kaas JH, De Carlos JA, Hevner RF, Lein E, Němec P. Evolution and development of the mammalian cerebral cortex. Brain Behav Evol. 2014;83: 126–139. 10.1159/000357753 24776993 PMC4440552

[3] Mo A, Mukamel EA, Davis FP, Luo C, Henry GL, Picard S, et al Epigenomic signatures of neuronal diversity in the mammalian brain. Neuron. 2015;86: 1369–1384. 10.1016/j.neuron.2015.05.018 26087164 PMC4499463

[4] Schroeder CE, Lindsley RW, Specht C, Marcovici A, Smiley JF, Javitt DC. Somatosensory input to auditory association cortex in the macaque monkey. J Neurophysiol. 2001;85: 1322–1327. 10.1152/jn.2001.85.3.1322 11248001

[5] Brett-Green B, Fifková E, Larue DT, Winer JA, Barth DS. A multisensory zone in rat parietotemporal cortex: intra- and extracellular physiology and thalamocortical connections. J Comp Neurol. 2003;460: 223–237. 10.1002/cne.10637 12687687

[6] Dehner LR, Keniston LP, Clemo HR, Meredith MA. Cross-modal circuitry between auditory and somatosensory areas of the cat anterior ectosylvian sulcal cortex: a “new” inhibitory form of multisensory convergence. Cereb Cortex. 2004;14: 387–403. 15028643 10.1093/cercor/bhg135

[7] Loeb GE, Fishel JA. Bayesian action & perception: Representing the world in the brain. Front Neurosci. 2014;8 10.3389/fnins.2014.00008 25400542 PMC4214374

[8] Stein BE, Stanford TR, Rowland BA. Development of multisensory integration from the perspective of the individual neuron. Nat Rev Neurosci. 2014;15: 520–535. 10.1038/nrn3742 25158358 PMC4215474

[9] Xu J, Bi T, Keniston L, Zhang J, Zhou X, Yu L. Deactivation of association cortices disrupted the congruence of visual and auditory receptive fields in superior colliculus neurons. Cereb Cortex. 2016;27: 5568–5578. 10.1093/cercor/bhw324 27797831

[10] Yu L, Xu J, Rowland BA, Stein BE. Multisensory plasticity in superior colliculus neurons is mediated by association cortex. Cereb Cortex. 2016;26: 1130–1137. 10.1093/cercor/bhu295 25552270 PMC4737606

[11] Budinger E, Heil P, Hess A, Scheich H. Multisensory processing via early cortical stages: Connections of the primary auditory cortical field with other sensory systems. Neuroscience. 2006;143: 1065–1183. 10.1016/j.neuroscience.2006.08.035 17027173

[12] Keniston LP, Henderson SC, Meredith MA. Neuroanatomical identification of crossmodal auditory inputs to interneurons in somatosensory cortex. Exp Brain Res. 2010;202: 725–731. 10.1007/s00221-010-2163-0 20087577 PMC2866510

[13] Zingg B, Hintiryan H, Gou L, Song MY, Bay M, Bienkowski MS, et al Neural networks of the mouse neocortex. Cell. 2014;156: 1096–1111. 10.1016/j.cell.2014.02.023 24581503 PMC4169118

[14] Henschke JU, Noesselt T, Scheich H, Budinger E. Possible anatomical pathways for short-latency multisensory integration processes in primary sensory cortices. Brain Struct Funct. 2015;220: 955–977. 10.1007/s00429-013-0694-4 24384580

[15] Bizley JK, Nodal FR, Bajo VM, Nelken I, King AJ. Physiological and anatomical evidence for multisensory interactions in auditory cortex. Cereb Cortex. 2007;17: 2172–2189. 10.1093/cercor/bhl128 17135481 PMC7116518

[16] Kayser C, Petkov CI, Augath M, Logothetis NK. Functional imaging reveals visual modulation of specific fields in auditory cortex. J Neurosci. 2007;27: 1824–1835. 10.1523/JNEUROSCI.4737-06.2007 17314280 PMC6673538

[17] Martuzzi R, Murray MM, Michel CM, Thiran J-P, Maeder PP, Clarke S, et al Multisensory interactions within human primary cortices revealed by BOLD dynamics. Cereb Cortex. 2007;17: 1672–1679. 10.1093/cercor/bhl077 16968869

[18] Zhou Y-D, Fuster JM. Somatosensory cell response to an auditory cue in a haptic memory task. Behav Brain Res. 2004;153: 573–578. 10.1016/j.bbr.2003.12.024 15265656

[19] Lemus L, Hernández A, Luna R, Zainos A, Romo R. Do sensory cortices process more than one sensory modality during perceptual judgments? Neuron. 2010;67: 335–348. 10.1016/j.neuron.2010.06.015 20670839

[20] Takagaki K, Zhang C, Wu J-Y, Lippert MT. Crossmodal propagation of sensory-evoked and spontaneous activity in the rat neocortex. Neurosci Lett. 2008;431: 191–196. 10.1016/j.neulet.2007.11.069 18178313 PMC2292672

[21] Sieben K, Röder B, Hanganu-Opatz IL. Oscillatory entrainment of primary somatosensory cortex encodes visual control of tactile processing. J Neurosci. 2013;33: 5736–5749. 10.1523/JNEUROSCI.4432-12.2013 23536087 PMC6705047

[22] Bieler M, Sieben K, Cichon N, Schildt S, Röder B, Hanganu-Opatz IL. Rate and temporal coding convey multisensory information in primary sensory cortices. eNeuro. 2017 10.1523/ENEURO.0037-17.2017 28374008 PMC5362936

[23] Komai S, Denk W, Osten P, Brecht M, Margrie TW. Two-photon targeted patching (TPTP) in vivo. Nat Protoc. 2006;1: 647–652. 10.1038/nprot.2006.100 17406293

[24] Iurilli G, Ghezzi D, Olcese U, Lassi G, Nazzaro C, Tonini R, et al Sound-driven synaptic inhibition in primary visual cortex. Neuron. 2012;73: 814–828. 10.1016/j.neuron.2011.12.026 22365553 PMC3315003

[25] McGinley MJ, David S V., McCormick DA. Cortical membrane potential signature of optimal states for sensory signal detection. Neuron. 2015;87: 179–192. 10.1016/j.neuron.2015.05.038 26074005 PMC4631312

[26] Ito J, Roy S, Liu Y, Cao Y, Fletcher M, Lu L, et al Whisker barrel cortex delta oscillations and gamma power in the awake mouse are linked to respiration. Nat Commun. 2014;5 10.1038/ncomms4572 24686563 PMC3988824

[27] Nishimura M, Sawatari H, Takemoto M, Song WJ. Identification of the somatosensory parietal ventral area and overlap of the somatosensory and auditory cortices in mice. Neurosci Res. 2015;99: 55–61. 10.1016/j.neures.2015.06.001 26068899

[28] Kheradpezhouh E, Adibi M, Arabzadeh E. Response dynamics of rat barrel cortex neurons to repeated sensory stimulation. Sci Rep. 2017;7 10.1038/s41598-017-11477-6 28904406 PMC5597595

[29] Pitas A, Albarracín AL, Molano-Mazón M, Maravall M. Variable temporal integration of stimulus patterns in the mouse barrel cortex. Cereb Cortex. 2017;27: 1758–1764. 10.1093/cercor/bhw006 26838770

[30] Brecht M, Roth A, Sakmann B. Dynamic receptive fields of reconstructed pyramidal cells in layers 3 and 2 of rat somatosensory barrel cortex. J Physiol. 2003;553: 243–265. 10.1113/jphysiol.2003.044222 12949232 PMC2343497

[31] Lee AK, Manns ID, Sakmann B, Brecht M. Whole-cell recordings in freely moving rats. Neuron. 2006;51: 399–407. 10.1016/j.neuron.2006.07.004 16908406

[32] Blanton MG, Lo Turco JJ, Kriegstein AR. Whole cell recording from neurons in slices of reptilian and mammalian cerebral cortex. J Neurosci Methods. 1989;30: 203–210. 2607782 10.1016/0165-0270(89)90131-3

[33] Margrie TW, Brecht M, Sakmann B. In vivo, low-resistance, whole-cell recordings from neurons in the anaesthetized and awake mammalian brain. Pflugers Arch. 2002;444: 491–498. 10.1007/s00424-002-0831-z 12136268

[34] Simons DJ. Multi-whisker stimulation and its effects on vibrissa units in rat SmI barrel cortex. Brain Res. 1983;276: 178–182. 6626997 10.1016/0006-8993(83)90561-9

[35] Wong-Riley M. Changes in the visual system of monocularly sutured or enucleated cats demonstrable with cytochrome oxidase histochemistry. Brain Res. 1979;171: 11–28. 223730 10.1016/0006-8993(79)90728-5

[36] Horikawa K, Armstrong WE. A versatile means of intracellular labeling: injection of biocytin and its detection with avidin conjugates. J Neurosci Methods. 1988;25: 1–11. 3146670 10.1016/0165-0270(88)90114-8

[37] Friedberg MH, Lee SM, Ebner FF. Modulation of receptive field properties of thalamic somatosensory neurons by the depth of anesthesia. J Neurophysiol. 1999;81: 2243–2252. 10.1152/jn.1999.81.5.2243 10322063

[38] Petersen CCH, Hahn TTG, Mehta M, Grinvald A, Sakmann B. Interaction of sensory responses with spontaneous depolarization in layer 2/3 barrel cortex. Proc Natl Acad Sci U S A. 2003;100: 13638–13643. 10.1073/pnas.2235811100 14595013 PMC263866

[39] Sachdev RNS, Ebner FF, Wilson CJ. Effect of subthreshold up and down states on the whisker-evoked response in somatosensory cortex. J Neurophysiol. 2004;92: 3511–3521. 10.1152/jn.00347.2004 15254074

[40] Van Looij MAJ, Liem SS, Van Der Burg H, Van Der Wees J, De Zeeuw CI, Van Zanten BGA. Impact of conventional anesthesia on auditory brainstem responses in mice. Hear Res. 2004;193: 75–82. 10.1016/j.heares.2004.02.009 15219322

[41] Cui J, Zhu B, Fang G, Smith E, Brauth SE, Tang Y. Effect of the level of anesthesia on the auditory brainstem response in the Emei Music Frog (Babina daunchina). PLOS ONE. 2017;12: e0169449 10.1371/journal.pone.0169449 28056042 PMC5215878

[42] Neimark MA, Andermann ML, Hopfield JJ, Moore CI. Vibrissa resonance as a transduction mechanism for tactile encoding. J Neurosci. 2003;23: 6499–6509. 12878691 10.1523/JNEUROSCI.23-16-06499.2003 PMC6740638

[43] Ghoshal A, Tomarken A, Ebner F. Cross-sensory modulation of primary sensory cortex is developmentally regulated by early sensory experience. J Neurosci. 2011;31: 2526–2536. 10.1523/JNEUROSCI.5547-10.2011 21325520 PMC6623684

[44] Ibrahim LA, Mesik L, Ji X ying, Fang Q, Li H fu, Li Y tang, et al Cross-modality sharpening of visual cortical processing through layer-1-mediated inhibition and disinhibition. Neuron. 2016;89: 1031–1045. 10.1016/j.neuron.2016.01.027 26898778 PMC4874809

[45] Wehr M, Zador AM. Balanced inhibition underlies tuning and sharpens spike timing in auditory cortex. Nature. 2003;426: 442–446. 10.1038/nature02116 14647382

[46] Drager UC, Hubel DH. Physiology of visual cells in mouse superior colliculus and correlation with somatosensory and auditory input. Nature. 1975;253: 203–204. 1110771 10.1038/253203a0

[47] Stein BE, Meredith MA. Multisensory integration. Neural and behavioral solutions for dealing with stimuli from different sensory modalities. Ann N Y Acad Sci. 1990;608: 51–70. 2075959 10.1111/j.1749-6632.1990.tb48891.x

[48] Kim SS, Gomez-Ramirez M, Thakur PH, Hsiao SS. Multimodal interactions between proprioceptive and cutaneous signals in primary somatosensory cortex. Neuron. 2015;86: 555–566. 10.1016/j.neuron.2015.03.020 25864632 PMC4409561

[49] Dooley JC, Franca JG, Seelke AMH, Cooke DF, Krubitzer LA. A connection to the past: Monodelphis domestica provides insight into the organization and connectivity of the brains of early mammals. J Comp Neurol. 2013;521: 3877–3897. 10.1002/cne.23383 23784751 PMC3959876

[50] Maunsell JH, Sclar G, Nealey TA, DePriest DD. Extraretinal representations in area V4 in the macaque monkey. Vis Neurosci. 1991;7: 561–573. 1772806 10.1017/s095252380001035x

[51] Zhou YD, Fuster JM. Visuo-tactile cross-modal associations in cortical somatosensory cells. Proc Natl Acad Sci U S A. 2000;97: 9777–9782. 10.1073/pnas.97.17.9777 10944237 PMC16941

[52] Jeffress LA. A place theory of sound localization. J Comp Physiol Psychol. 1948;41: 35–39. 18904764 10.1037/h0061495

[53] Hutson KA, Masterton RB. The sensory contribution of a single vibrissa’s cortical barrel. J Neurophysiol. 1986;56: 1196–1223. 10.1152/jn.1986.56.4.1196 3783236

[54] Goto K, Ueki A, Iso H, Morita Y. Involvement of nucleus accumbens dopaminergic transmission in acoustic startle: observations concerning prepulse inhibition in rats with entorhinal cortex lesions. Psychiatry Clin Neurosci. 2004;58: 441–445. 10.1111/j.1440-1819.2004.01281.x 15298660

[55] Hopfinger JB, Mangun GR. Tracking the influence of reflexive attention on sensory and cognitive processing. Cogn Affect Behav Neurosci. 2001;1: 56–65. 12467103 10.3758/cabn.1.1.56

[56] O’Donnell BF, Vohs JL, Hetrick WP, Carroll CA, Shekhar A. Auditory event-related potential abnormalities in bipolar disorder and schizophrenia. Int J Psychophysiol. 2004;53: 45–55. 10.1016/j.ijpsycho.2004.02.001 15172135

[57] Hurlbut BJ, Lubar JF, Satterfield SM. Auditory elicitation of the P300 event-related evoked potential in the rat. Physiol Behav. 1987;39: 483–487. 3575494 10.1016/0031-9384(87)90377-5

[58] Ehlers CL, Somes C. Long latency event-related potentials in mice: effects of stimulus characteristics and strain. Brain Res. 2002;957: 117–128. 12443987 10.1016/s0006-8993(02)03612-0

[59] Cote KA, Campbell KB. The effects of varying stimulus intensity on P300 during REM sleep. Neuroreport. 1999;10: 2313–2318. 10439455 10.1097/00001756-199908020-00017

[60] Yamaguchi S, Knight RT. P300 generation by novel somatosensory stimuli. Electroencephalogr Clin Neurophysiol. 1991;78: 50–55. 1701715 10.1016/0013-4694(91)90018-y

[61] Valeriani M, Fraioli L, Ranghi F, Giaquinto S. Dipolar source modeling of the P300 event-related potential after somatosensory stimulation. Muscle Nerve. 2001;24: 1677–1686. 11745977 10.1002/mus.1203

