# Supplementary figures and images for: Auditory-induced response in the primary sensory cortex of rodents
Source: PLoS One. 2018 Dec 20;13(12):e0209266. doi: 10.1371/journal.pone.0209266 (PMC6301624; doi:10.1371/journal.pone.0209266)

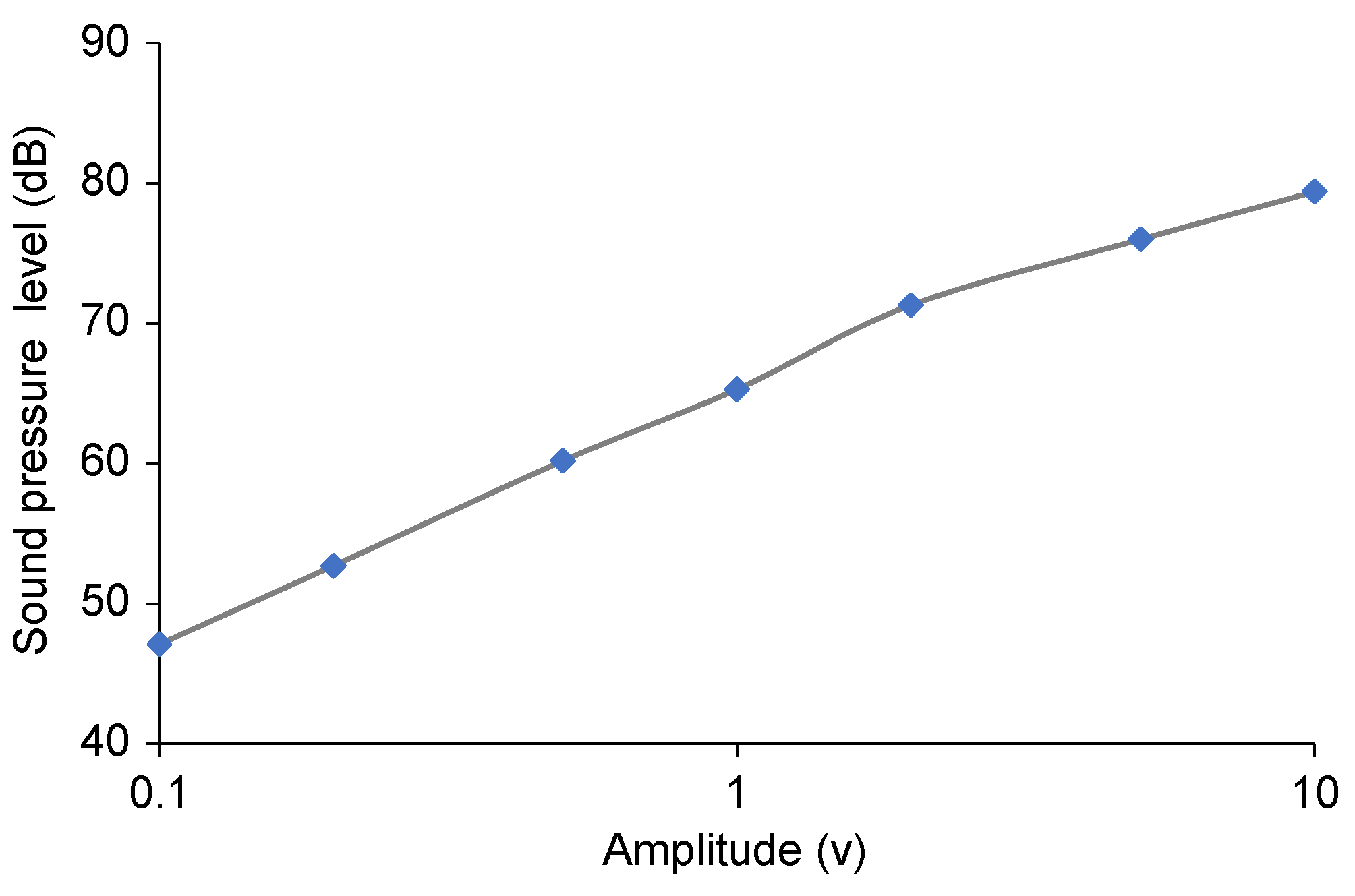

Supplement: S1 Fig — The relationship between amplitude of the operated stimulation and the recorded sound pressure level with the detector is indicated. Abscissa is shown with a logarithmic scale. (TIF) [file pone.0209266.s001.tif]
